# Rural-urban comparisons of dengue seroprevalence in Malaysia

**DOI:** 10.1186/s12889-016-3496-9

**Published:** 2016-08-18

**Authors:** Cheng Hoon Chew, Yuan Liang Woon, Faridah Amin, Tassha H. Adnan, Asmah Hani Abdul Wahab, Zul Edzhar Ahmad, Mohd Adam Bujang, Abdul Muneer Abdul Hamid, Rahman Jamal, Wei Seng Chen, Chee Peng Hor, Lena Yeap, Ling Ping Hoo, Pik Pin Goh, Teck Onn Lim

**Affiliations:** 1 Clinical Research Centre, c/o Third Floor, Dermatology Block, Hospital Kuala Lumpur, Jalan Pahang, 50586 Kuala Lumpur, Malaysia; 2 National Public Health Laboratory, Lot 1853, Kg. Melayu, 47000 Sungai Buloh, Selangor Kuala Lumpur, Malaysia; 3 UKM Medical Molecular Biology Institute, UKM Medical Centre, Jalan Yaacob Latiff, Bandar Tun Razak, 56000 Cheras Kuala Lumpur, Malaysia; 4 Klinik Alam Medic, 41, Jalan Perdana ¾, Taman Puchong Perdana, 47100 Puchong, Selangor Kuala Lumpur, Malaysia; 5 Kepala Batas Hospital, Jalan Bertam 2, 13200 Kepala Batas, Penang Malaysia; 6 Stats Consulting Pte Ltd, D7-3-1, Block D7, Pusat Perdagangan Dana 1, Jalan PJU 1A/46, PJU 1A, 47301 Petaling Jaya, Selangor Malaysia; 7 ClinResearch Pte Ltd, D7-3-1, Block D7, Pusat Perdagangan Dana 1, Jalan PJU 1A/46, PJU 1A, 47301 Petaling Jaya, Selangor Malaysia

**Keywords:** Dengue, Seroprevalence, Urban rural difference, Malaysia

## Abstract

**Background:**

Each year an estimated 390 million dengue infections occur worldwide. In Malaysia, dengue is a growing public health concern but estimate of its disease burden remains uncertain. We compared the urban-rural difference of dengue seroprevalence and determined age-specific dengue seroprevalence in Malaysia.

**Methods:**

We undertook analysis on 11,821 subjects from six seroprevalence surveys conducted in Malaysia between 2001 and 2013, which composed of five urban and two rural series.

**Results:**

Prevalence of dengue increased with age in both urban and rural locations in Malaysia, which exceeded 90 % among those aged 70 years or beyond. The age-specific rates of the 5 urban surveys overlapped without clear separation among them, while prevalence was lower in younger subjects in rural series than in urban series, the trend reversed in older subjects. There were no differences in the seroprevalence by gender, ethnicity or region. Poisson regression model confirmed the prevalence have not changed in urban areas since 2001 but in rural areas, there was a significant positive time trend such that by year 2008, rural prevalence was as high as in urban areas.

**Conclusion:**

Dengue seroprevalence has stabilized but persisted at a high level in urban areas since 2001, and is fast stabilizing in rural areas at the same high urban levels by 2008. The cumulative seroprevalence of dengue exceeds 90 % by the age of 70 years, which translates into 16.5 million people or 55 % of the total population in Malaysia, being infected by dengue by 2013.

## Background

Dengue is an infection caused by the arbovirus that belongs to the genus *Flavivirus* (family Flaviviridae) and comprises four distinct, but closely related serotypes, *DENV 1,2,3* and *4* [1]. Infection with any serotype confers lifelong immunity to that serotype, with partial and temporary cross immunity to other serotypes [1, 2]. The dengue virus is an arthropod-borne virus that is primarily transmitted to humans by the *Aedes aegypti* and *Aedes albopictus* mosquitoes, a highly competent vector that is well adapted to human modified environment. It has spread extensively throughout the tropics and sub-tropics during the past 200 years through shipping trade routes. The spread of the vector and hence the infection were amplified during the Second World War through the huge increase in movement of people and equipment aided by modern transportation [1, 3–5].

Following the Second World War, rapid expansion and urbanization of the populations in South East Asia (SE Asia) and other tropical countries further intensified dengue transmission [3, 4]. Many dengue outbreaks have been documented since the 1950s in SE Asia and this has continued in ever larger cyclical epidemics [4]. By 2012, WHO reported that global incidence has increased 30-fold in the past 50 years and estimated that some 50 – 100 million new infections occurred annually [6]. A recent estimate using the cartographic approach has pushed this number up to 390 million infections a year, more than three times WHO’s estimate and Asia bore a disproportionate share of 70 % of the global dengue burden [7]. In SE Asia, recent estimates have also shown a continuing increase in dengue incidence [8]. For example, in Malaysia where all 4 serotypes are circulating, the most recent estimate showed that incidence of dengue has risen 7 fold over the period between 2000 and 2010 [9]. These estimates of the increasing dengue incidence were based on data from dengue surveillance and cohort studies. Estimating incidence based on notification data is unreliable because of variable surveillance systems and healthcare infrastructures within and between countries. Besides, in contrast to infrequent sporadic infection, for a hyper-endemic infection like dengue which had infected more than half of the population at any one time, and more than 90 % of them over their lifetimes, notification of 35 million or more people over a decade or more in a typical mid-sized population like Malaysia poses significant practical challenges. The pre-existing passive dengue surveillance system is good for monitoring general trends, but inadequate to obtain a precise number of dengue cases as the total number of dengue cases is usually underreported in passive surveillance. For cohort studies, obtaining a sizable representative sample of both children and adults nationwide and following them over their lifetimes to ascertain infection risk is no less challenging especially in the developing countries where dengue is endemic. We believe that repeated seroprevalence surveys using low cost IgG enzyme-linked immunosorbant assays (ELISAs) is the most practical and likely most reliable method to estimate dengue incidence in endemic countries, though such surveys were uncommonly used in dengue epidemiologic research. A recent systematic review identified only 53 published seroprevalence surveys between 1980 and 2010 worldwide, and of these, only 30 surveys from 18 countries had usable IgG data and only one country had serial sero-survey data from multiple years [10]. Seroprevalence data provide estimates of the proportion of people in a population who have been previously infected by dengue. Series of seroprevalence data over several years also provides the basis for estimating disease incidence based on mathematical modelling of the relationship between incidence, prevalence and mortality. This approach has been widely used in HIV [11] and malaria [12] epidemiological research to estimate incidence and infection burden in endemic countries, but to our knowledge rarely used in the dengue research. This study aimed to compare the urban-rural difference of dengue seroprevalence and to determine age-specific dengue seroprevalence in Malaysia.

## Methods

This study performed trend analysis on data extracted and compiled from published articles on dengue seroprevalence [13–15] and unpublished data collected from the sentinel surveillance conducted by National Public Health Laboratory (NPHL) of the Ministry of Health (MOH), Malaysia [16]. All patient data were analysed anonymously.

### Ethical approval

This study was registered under National Medical Research Registry (NMRR, NMRR-15-961-26546) and was approved by Medical Research Ethics Committee, Ministry of Health.

### Study population

Table 1 outlines the demographic characteristics for the 6 studies on dengue seroprevalence as mentioned above. All the studies included Malaysians from all age groups with exception of the Malaysian Cohort (TMC) study which included adults aged between 35 and 74 years. All studies were urban based except 2 series, Abu Bakar S. and Lim YA (ABL) which was entirely rural and TMC which has 40 % of its sample from rural areas.

**Table 1:** Demographic characteristics of dengue seroprevalence studies in Malaysia

Data source	^a^Chen et al.	^b^Abu Bakar S. and Lim YA (ABL)	^c^The Malaysian Cohort study (TMC)	^d^NPHL	^d^NPHL	^d^NPHL
Year of study conducted	2001	2007	2008	2011	2012	2013
Study setting	Private primary care clinic	School and Community	Community based nationwide study	Public primary care clinics	Public primary care clinics	Public primary care clinics
Sample size	85	1800	1000	3762	3911	1263
Mean age (in years)	43	16	53	39	35	38
Age range (in years)	5 - 71	7 – 49	35-74	1- 93	1- 88	1- 90
Gender (%)						
Female	46	No data	60	56	55	55
Male	54	No data	40	44	45	45
Ethnicity (%)						
Malay	8	No data	57	48	63	40
Chinese	70	No data	37	27	15	25
Indian & others	22	No data	6	25	22	35
Geographical area (%)						
Peninsular Malaysia	100	100	100	87	87	51
East Malaysia	0	0	0	13	13	49
Location (%)						
Urban	100	0	60	100	100	100
Rural	0	100	40	0	0	0

^a^Suburban community in Puchong; ^b^Aboriginal communities throughout the countries; ^c^National cohort study throughout the country; ^d^7 primary care clinics all located at city areas in different states of Selangor, Wilayah Persekutuan Kuala Lumpur, Perak, Kelantan, Johor and Sabah

Study by Chen et al. [14] was based at a single urban primary care clinic where blood specimens of patients who attended for reasons unrelated to dengue or other febrile illnesses were collected [14]. The ABL was a rural school and community based study. TMC study was a nationwide, community based study of adults aged 35–74 years living in urban and rural areas [17]. The NPHL conducts regular sentinel surveillance based at public health clinics located in urban areas only. In the surveillance, blood specimen of patients who attended the health clinics for reasons unrelated to dengue or other febrile illness from the age of 1 year old onward was collected (Amin F., personal communication). None of the studies included were hospital based, nor were blood specimens collected primarily for another purpose. However, none of the surveys were based on a random sample of households recruited from throughout the country. We obtained individual level records for all the studies except the ABL study.

We were unable to include data from two dengue vaccine trials conducted among Malaysian children [18, 19], because we have no access to the data and the studies employed a different serology method, which was serotype-specific plaque reduction neutralisation test (PRNT_50_).

### Serological test

The presence of dengue IgG antibodies in sera samples for all the seroprevalence studies was detected using the PanBio dengue IgG indirect ELISA [13–16].

### Statistical analysis

#### Age-specific seroprevalence rates of dengue

The age-specific seroprevalence rate was constructed using the cubic spline method [20] by using Stata Version 13. The method interpolated prevalence rates specified per five-year interval (or other irregular intervals) to one-year age groups.

### Poisson regression model

Poisson regression model with random effect was used to determine time trend in dengue seroprevalence and age dependency in the risk of dengue infection stratified by urban or rural location. The outcome variable was count of seroconverted subjects while each study included was treated as random effect. The fixed effects were calendar year, age group and urban or rural location. The model equation is: Log (P_i_) = Log(N_i_) + β_0_ + β_1_ Age + β_2_ Year + β_3_ Urban-Rural (UR) location + β_4_Age_is_*Year + β_5_Age*UR + β_6_ Year*i.UR + μ_s_ + ε_is_, where P_i_ is the Prevalent count of dengue of a group i, which is cross-classified by levels of Age (8 levels) and UR location (2 levels). Log(N) is the log transformed sample size of each Age-UR groups, β_0_ is a constant, β is a slope coefficient, μ_s_ is the between survey error and ε_is_ is the within group error.

Interactions were included in the Poisson regression model using age-by-calendar year, calendar year-by-location and location-by-age. We hypothesised that: 1) when the age-by-calendar year interaction was positive and significant, seroprevalence of dengue increased with age and time; 2) when the calendar year-by-location interaction was positive and significant, seroprevalence of dengue increased over time and was dependent on urban-rural difference; 3) when the age-by-location interactions was positive and significant, seroprevalence of dengue increased with age of subjects and was dependent on urban-rural difference. Likelihood Ratio test for nested models or Bayesian Information Criterion for non-nested models was used to find the best fit for the data. The best fitting model is used to derive the pooled estimates of age-specific seroprevalence rates.

## Results

Seroprevalence data were available for pooled sample of 11,821 subjects. The rate of seroprevalence in urban locations ranged between 61 and 92 % and in rural locations ranged between 28 and 91 %. (Table 2).

**Table 2:** Demographic characteristics and seroprevalence of dengue in Malaysia

Study	Chen	ABL	TMC	NPHL	NPHL	NPHL
Year	2001	2007	2008	2011	2012	2013
Sample size	85	1800	1000	3762	3911	1263
Overall crude seroprevalence (%)	77	20	92	65	61	61
Gender (%)						
Male	74	No data	94	66	64	60
Female	79	No data	90	64	59	61
Ethnicity (%)						
Malay	88	No data	92	56	59	55
Chinese	71	No data	91	76	68	66
Indian and others	No data	No data	92	68	60	64
Geographical area (%)						
Peninsular Malaysia	77	20	94	65	60	57
East Malaysia	No data	No data	No data	58	61	65
Location (%)						
Urban	77	No data	92	65	61	61
Rural	No data	20	91	No data	No data	No data

**Fig. 1:**
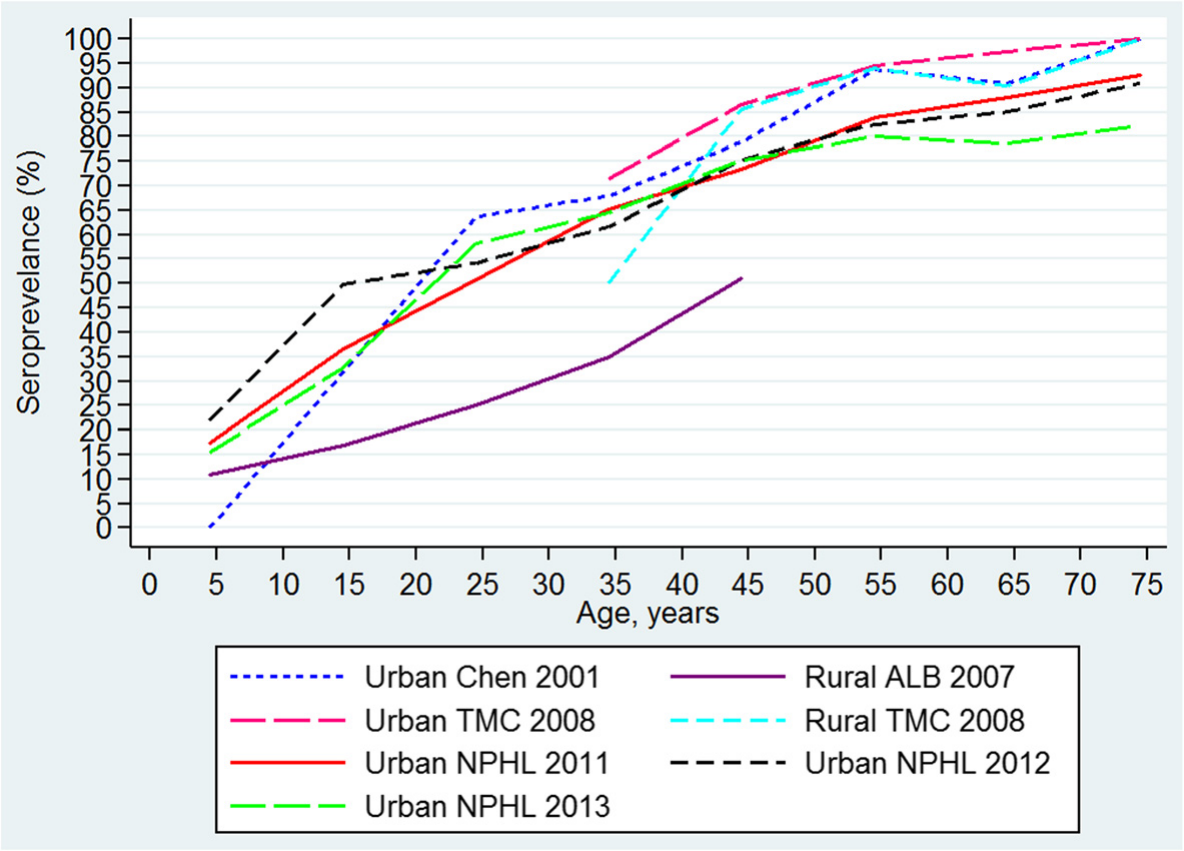
Age-specific seroprevalence of dengue observed between 2000 and 2013

The observed crude age-specific seroprevalence derived from the 6 studies were plotted as illustrated in Fig. 1 shows cubic spline smoothed estimates of age-specific seroprevalence for each study (TMC’s rates below the age 35 and ABL’s rates above age 49 were extrapolated from available data in other age groups). The 5 urban studies have similar seroprevalence profiles by age. Seroprevalence rate of dengue increased with age in both urban and rural locations. By 65 years of age, at least 80 % of Malaysian populations from both urban and rural locations were infected with dengue.

**Fig. 2:**
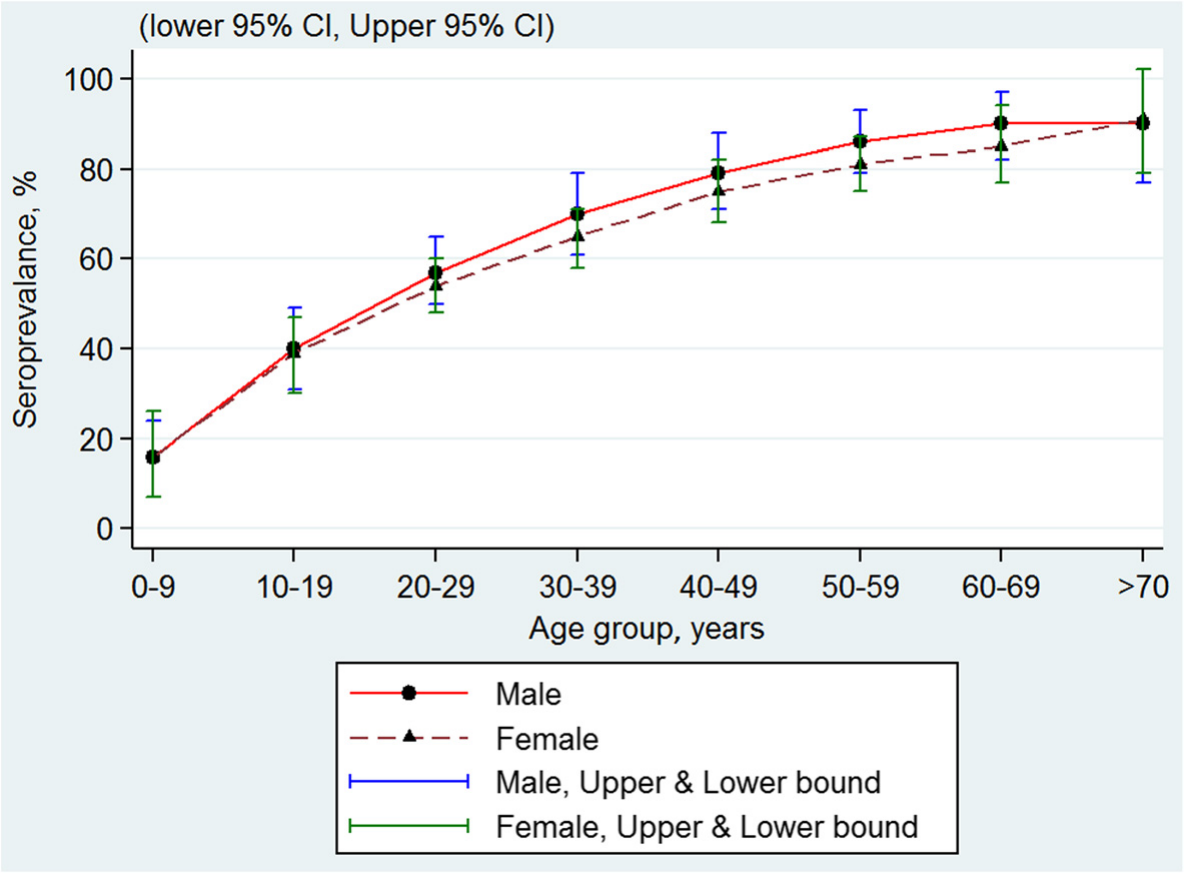
Age-specific dengue seroprevalence estimates* by gender. Cubic spline smoothed and pooled across all surveys

**Fig. 3:**
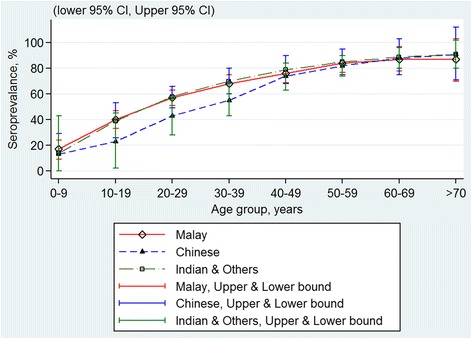
Age-specific dengue seroprevalence estimates* by ethnicity. Cubic spline smoothed and pooled across all surveys

**Fig. 4:**
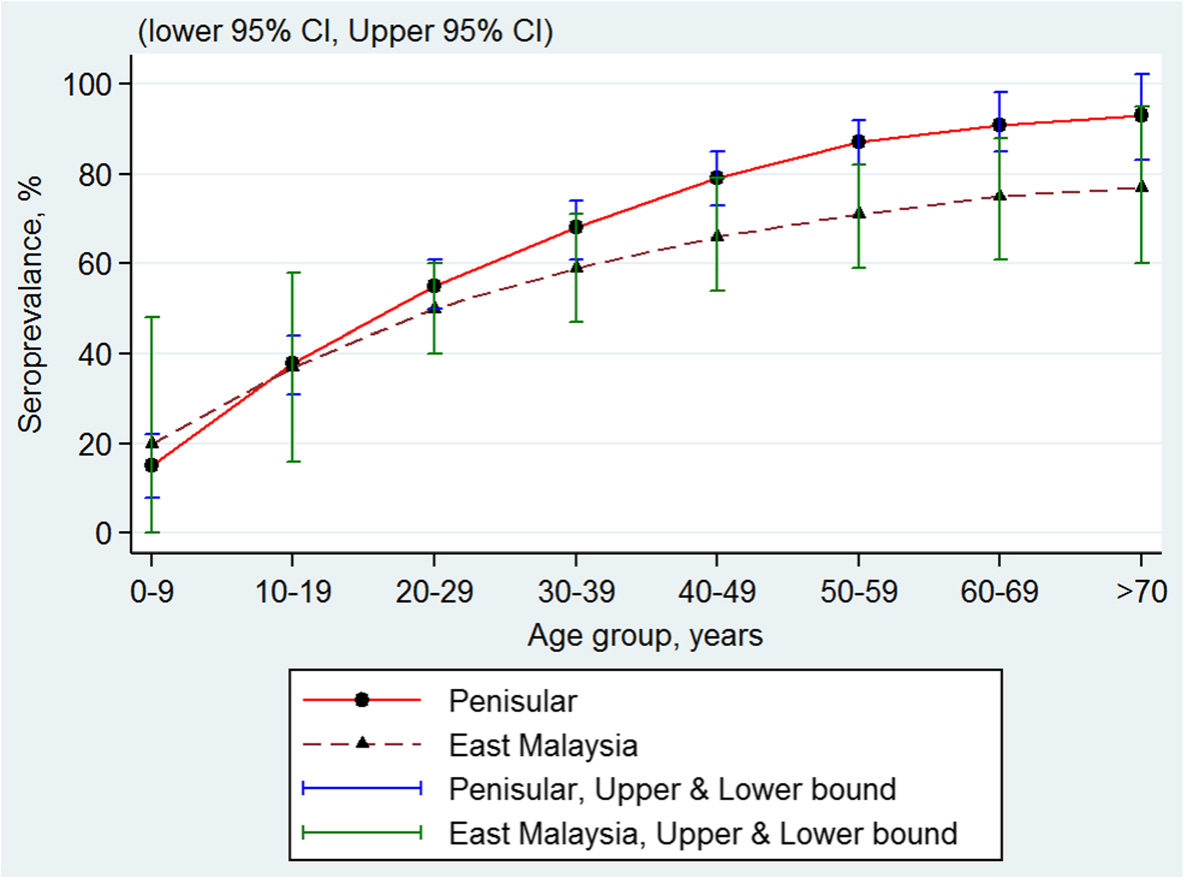
Age-specific dengue seroprevalence estimates* by region. Cubic spline smoothed and pooled across all surveys

**Fig. 5:**
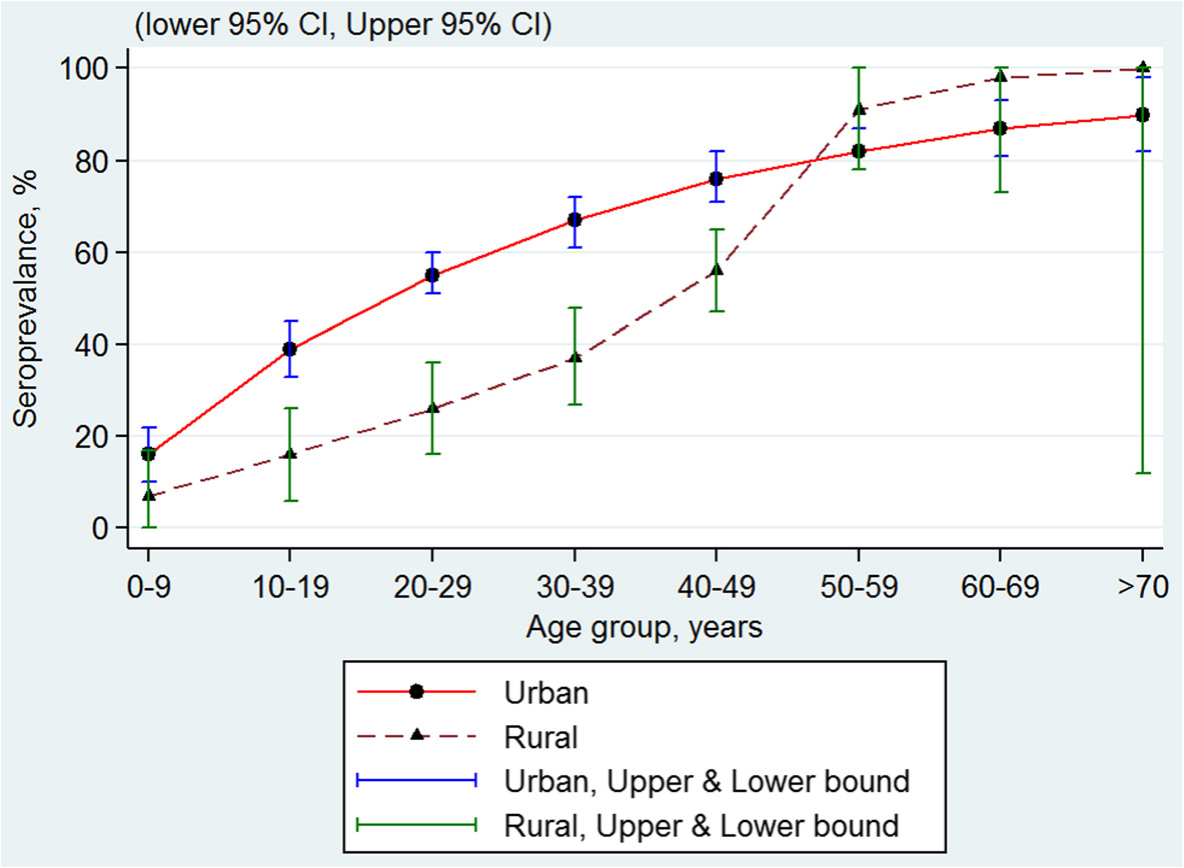
Age-specific dengue seroprevalence estimates* by urban-rural location. Cubic spline smoothed and pooled across all surveys

Using pooled data, there were no significant differences in the seroprevalence with regards to gender (Fig. 2), ethnicity (Fig. 3) and geographical areas (Fig. 4) across all age groups, as their 95 % CI overlapped with each other. The age-specific rate in urban areas was higher among the younger age of less than 40 years as compared to those from rural locations. The age-specific rate in urban areas was higher among the younger population aged of less than 40 years as compared to those from rural areas, whereas this trend is reversed by the age of 40 years, though the 95 % confidence intervals overlapped at the higher age range (Fig. 5).

**Fig. 6:**
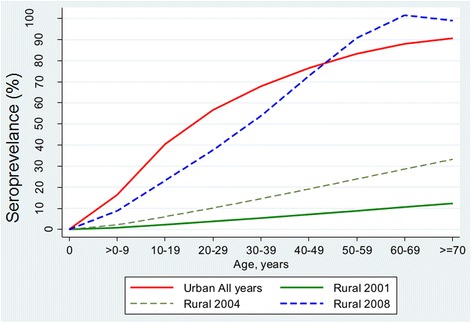
Predicted age-specific dengue seroprevalence rate using Poisson regression model by urban-rural locations

The Poisson regression model shows a statistically significant increase in dengue seroprevalence rates with increasing age (Table 3), higher among those living in urban locations as compared to rural locations (Fig. 6).

**Table 3:** Summary of regression coefficient (95 % CI and p-values) from Poisson model of dengue seroprevalence in Malaysia between 2001 and 2013

Variable	Coefficient	95 % CI	*p*-value
Age group (in year)			
0–9	0		-
10–19	0 · 88	(0 · 70, 1 · 07)	<0 · 0001
20–29	1 · 24	(1 · 07, 1 · 41)	<0 · 0001
30–39	1 · 42	(1 · 25, 1 · 60)	<0 · 0001
40–49	1 · 56	(1 · 39, 1 · 73)	<0 · 0001
50–59	1 · 63	(1 · 46, 1 · 80)	<0 · 0001
60–69	1 · 69	(1 · 51, 1 · 86)	<0 · 0001
> 70	1 · 73	(1 · 55, 1 · 91)	<0 · 0001
Location			
Urban (referent)	0	-	-
Rural	-600.91	(-1042, -160)	0 · 0008
Calendar year	-0 · 0009	-	<0 · 0001
Calendar year-by-Location interaction			
All years at Urban location (referent)	0	-	-
All years at Rural location	0 · 30	(0 · 08, 0 · 52)	0 · 0008
Age-by-Location interaction			
All ages at Urban location (referent)	0	-	-
Age 0–9 at Rural location	0	-	-
Age 10–19 at Rural location	-0 · 07	(-0 · 49, 0 · 35)	0 · 742
Age 20–29 at Rural location	0 · 06	(-0 · 41, 0 · 54)	0 · 799
Age 30–39 at Rural location	0 · 24	(-0 · 21, 0 · 68)	0 · 294
Age 40–49 at Rural location	0 · 45	(0 · 04, 0 · 86)	0 · 031
Age 50–59 at Rural location	0 · 58	(0 · 18, 0 · 98)	0 · 004
Age 60–69 at Rural location	0 · 67	(0 · 26, 1 · 07)	0 · 001
Age >70 at Rural location	0 · 81	(0 · 40, 1 · 23)	<0 · 0001
Calendar year-by-Age interaction	-0 · 003 to 0 · 05	-	0 · 06 to 0 · 9

When interactions were included in the model, there is a significant difference in the time trends of dengue seroprevalence by location (calendar year-by-location interaction) (Table 3). While there has been no change in dengue prevalence at all in urban locations over time, dengue prevalence at rural locations increased over the years, showing a significant positive time trend. The age-by-location interaction is also significant suggesting that there is in addition an age dependent difference between urban and rural locations. The interaction between calendar year (time) and age is not significant (all *p*-values > 0.4) indicating the age dependency in dengue seroprevalence has not changed over time.

Figure 6 shows the predicted age-specific dengue seroprevalence based on the results of the Poisson model (Table 3). In the absence of time trend (changes over calendar year), the data from predicted age-specific seroprevalence in urban locations for all years were pooled and presented in a single line. On the other hand, seroprevalence rates in rural areas show both positive time trend and age dependency. Thus, predicted age-specific seroprevalence was analysed and plotted for each calendar year. Only data for years 2001, 2004 and 2008 were presented here to avoid crowding of Fig. 6. The slopes of each of the 3 rural smoothened lines and the single urban line differ from one another indicating age dependency.

## Discussion

### Time trend in dengue seroprevalence

The increase in seroprevalence of dengue with age provides a measure of dengue infection in the past. The high seroprevalence observed since 2001 indicates a continuous high level of dengue virus exposure in our population. The age-specific dengue seroprevalence in age *x* is a measure of cumulative prevalence of dengue at age *x*. However a single prevalence series cannot distinguish between no change in past dengue risk from time and/or age-dependent change in risk of exposure. Our results, based on multiple series observed between 2001 and 2013, showed neither a time trend nor significant age dependency in dengue seroprevalence in urban area. In other words, the dengue seroprevalence has been uniformly high in all age groups in urban areas in Malaysia since year 2001.

### Urban-rural difference in dengue seroprevalence

Dengue is a highly transmissible urban disease on account of its mosquito vector, *Aedes aegypti,* which is well adapted to urban human environments [21]. The risk differential between urban and rural areas reported here is expected, especially in the younger age groups. Malaysia GDP per capita has increased 2.7 fold from USD 4,000 to USD 11,000 between 2001 and 2014 [22]. Besides, the urban population in Malaysia has increased from 62 % of total population in 2000 to 71 % in 2010 [23]. This rapid economic growth in the past 15 years and the associated increasing urbanization, as well as increasing population mixing enabled by the modern transportation network in the country all might have contributed to increasing homogeneity in dengue risk over time between urban and rural areas and in all age groups. Between 2001 and 2008, as shown in this study, the prevalence of dengue in rural areas was rising fast and converging towards the high levels observed in the urban population. We caution however that the prevalence estimates reported here for rural areas are based on only a small sample (*n* = 2200 out of total sample of 11,821 subjects) from 2 sero-surveys and may be unduly sensitive to the limited data, as compared to 5 sero-surveys for urban areas. To our knowledge, 30 sero-surveys were conducted in 18 countries worldwide over the past 3 decades [10], but none of them reported on urban-rural differences to allow us to compare our results. We acknowledge that if another much larger survey had been conducted in the past in this or other country, it may not necessary show any difference between urban and rural locations.

This study has excluded data from two dengue vaccine trials which had included Malaysian children on account of the use of serotype-specific PRNT_50_ test [18, 19]. These trials have reported a very high baseline seroprevalence rate of 68 % among the participating children aged 2 – 14, which is much higher than the 35 % rate in urban children by age 14 reported in this study. This is likely due to the use of highly sensitive PRNT_50_ test in these trials. Hence, the use of the less sensitive ELISA IgG test in the six sero-surveys included in this study has led us to underestimate the dengue seroprevalence. The actual seroprevalence in Malaysia was likely to be higher than the already very high level reported here.

### Dengue notification, healthcare utilization and disease severity

There has been undoubtedly an increase in dengue notification in the past 15 years [24]. The number notified reached an all-time high of 108, 698 cases in 2014 (http://idengue.remotesensing.gov.my/idengue/index.php). This has been widely interpreted as reflecting an underlying increasing trend in dengue incidence [8, 9] but our finding of a stable but high infection risk since 2001 is not compatible with this interpretation. Similarly the apparent age shift in dengue notification rates [25], widely interpreted as showing a shift in the risk of dengue to older age groups, is not compatible with our finding of uniform risk across all age groups in urban areas. We hypothesize that this paradoxical phenomenon of increasing notification and yet unchanging risk since 2001 is better explained by increasing healthcare utilization for dengue illness. In 2014, dengue was the top reason for hospitalization accounting for 14 % of hospital admissions, and top 23rd reason for visits to primary care clinics in Malaysia [26]. Besides the increasing availability and wider distribution of health services, this may be partly driven by increasing number of symptomatic and severe cases especially in the older age groups, in contrast to the past observation that severe disease is predominantly seen in pediatric population [27]. This age shift in disease severity in turn may be related to the high persistent background dengue risk in this population leading to higher risk of secondary infections with prolonged exposure, which correlates with disease severity.

### Estimation of dengue incidence

The demonstration of absence of time trend and age dependency in dengue risk in urban locations provides the justification for using a single pooled age-specific seroprevalence rates to estimate the age-specific incidence of dengue in urban population in Malaysia. For rural population however, age- and year-specific estimates will be required for the purpose. This is the subject of an ongoing work to estimate the incidence of dengue, incidence of dengue notification and hospitalization, as well as excess mortality associated with dengue illness.

## Conclusions

The increasing incidence of dengue, often occurring in cyclical epidemics, has been well documented and it is a major public health concern in Malaysia. The trend analysis reported here showed that the risk of dengue has stabilized but persisted at a high level in urban areas since 2001, and is fast stabilizing in rural areas at the same high urban levels by year 2008. In the Malaysian population, the prevalence of infection is conservatively estimated to exceed 90 % by the age of 70 years. This translates into 16.5 million people, or 55 % of total Malaysian population, who have previously been infected by dengue by 2013.
